# Case Report: A minimally disruptive technique for the management of frozen leads

**DOI:** 10.3389/fcvm.2025.1518177

**Published:** 2025-02-11

**Authors:** Su-Huan Chang, Kuan-You Lin, Chih-Chieh Yu

**Affiliations:** ^1^ Department of Internal Medicine, College of Medicine, National Taiwan University, Taipei, Taiwan; ^2^ Division of Cardiology, Department of Internal Medicine, National Taiwan University College of Medicine and Hospital, Taipei, Taiwan

**Keywords:** electrophysiology, pacemaker generator replacement, frozen lead, silicone plug, setscrew

## Abstract

During pacemaker generator replacement, difficulties may arise with the set screw not being properly engaged, a situation referred as a “frozen lead”. We reported a case of an 88-year-old woman with a 15-year-old generator and increasing impedance of the right atrial lead. The disruption of the silicone plug and removal of the hidden metal ring facilitated successful disconnection of the generator without damaging the lead. Postoperative assessments demonstrated normalized right atrial lead impedance and stable parameters at follow-up. This case highlights the effectiveness of a minimally invasive approach to managing frozen leads, providing valuable insights in similar clinical scenarios.

## Introduction

With the extension of life expectancy due to advancements in public health measures and medical care, the incidence of cardiac implantable electronic devices (CIED) implantation has steadily increased over the past 50 years. As a result, the frequency of generator replacement procedures has also risen significantly, particularly due to end-of-service or other factors, becoming a more common procedure in electrophysiology laboratories. The leads are securely connected to the pulse generator head using a screw-in method that requires a universal screwdriver. During a pulse generator replacement procedure, the leads must first be unscrewed and disconnected from the old generator before being attached to a new one. Unfortunately, it is not always easy to unscrew and disconnect all leads—a situation referred to as “frozen lead”. In such cases where the leads may become stuck in the connector and cannot be removed, several approaches have been described until now. Report on usage of orthopedic electrical drill to create a channel through the plastic head of the generator was published previously (1). Erne et al. have attempted to facilitate lead disconnection using specific solvents such as ethanol or dimethyl sulfoxide (2). Setscrew damage is thought to be the most common cause of “frozen lead”. Recently, a simple method using an aseptic latex glove to enhance torque while unscrewing the lead was reported (3). Since there is no established standard approach for this issue, we present our case and introduce a simple first step to address this problem.

## Case presentation

An 88-year-old female patient was scheduled for a generator replacement due to battery depletion. She had undergone implantation of an Abbott DDDR pacemaker for sick sinus syndrome 15 years ago. The generator was an Identity ADx XL DR 5386, and the RA and RV leads were IsoFlex® S (Models 1642T and 1646T, respectively). Her follow-up course had been uneventful, with the exception of a gradual increase in the bipolar right atrial (RA) lead impedance, rising from approximately 900 ohms to 1,600 ohms over the past year. Prior to surgery, alternative management strategies were thoroughly discussed with the patient, including the implantation of a new RA lead with or without lead extraction or retaining the existing lead in place.

**Figure 1:**
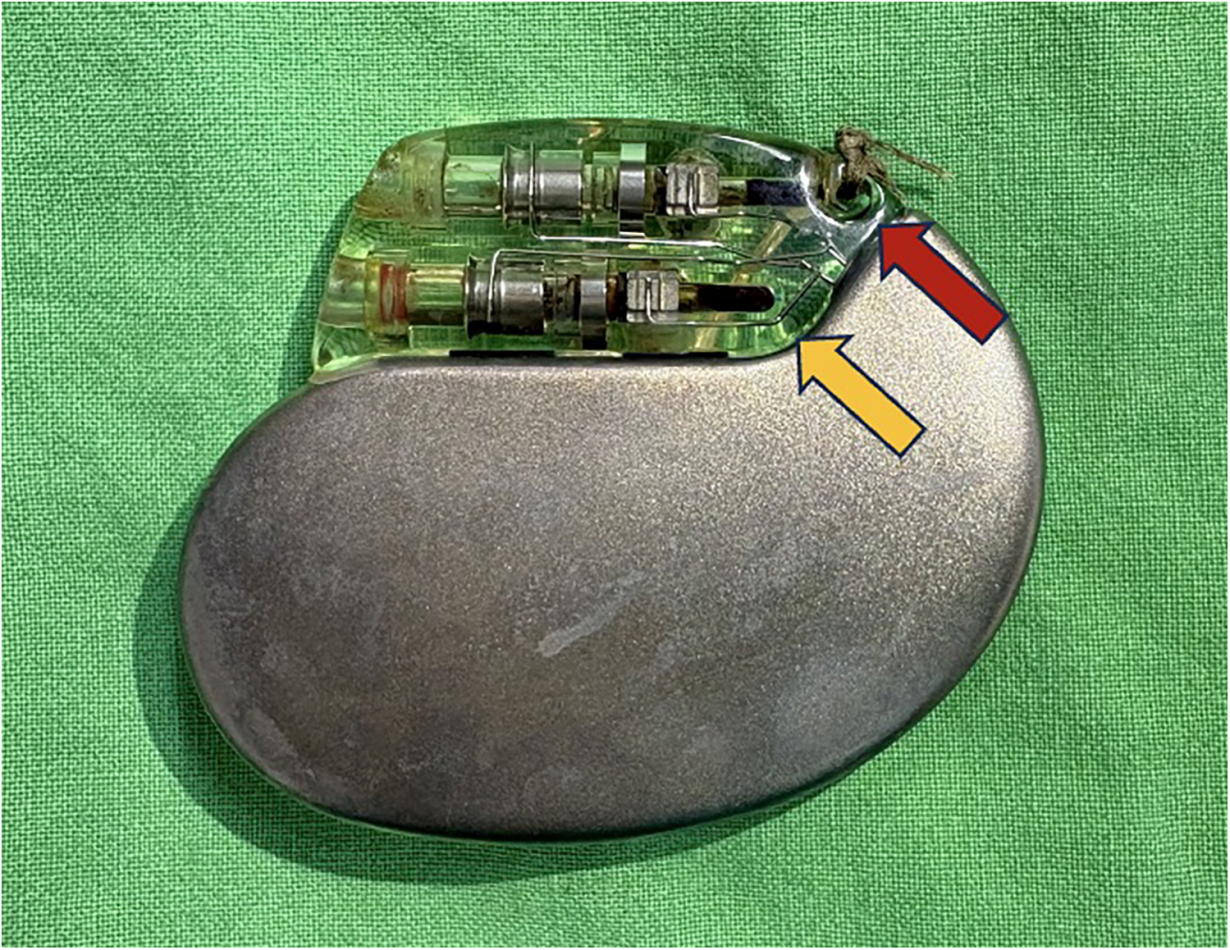
The dark brown discoloration of the RA (red arrow) and RV (yellow arrow) pin ports.

In the operating room, following a skin incision and dissection of the surrounding tissue and capsule, the old pulse generator was successfully extracted. Upon inspection, an unusual dark brown discoloration was noted in both pin ports (Figure 1). While the RV lead was easily unscrewed, difficulty was encountered with the RA lead. The screwdriver was unable to properly engage the set screw through the silicone plug, the mechanism of which was unclear. Despite attempts to manipulate the screw from various angles and cleaning the hexagonal socket with fine tweezers and a needle, the screw remained inaccessible.

**Figure 2:**
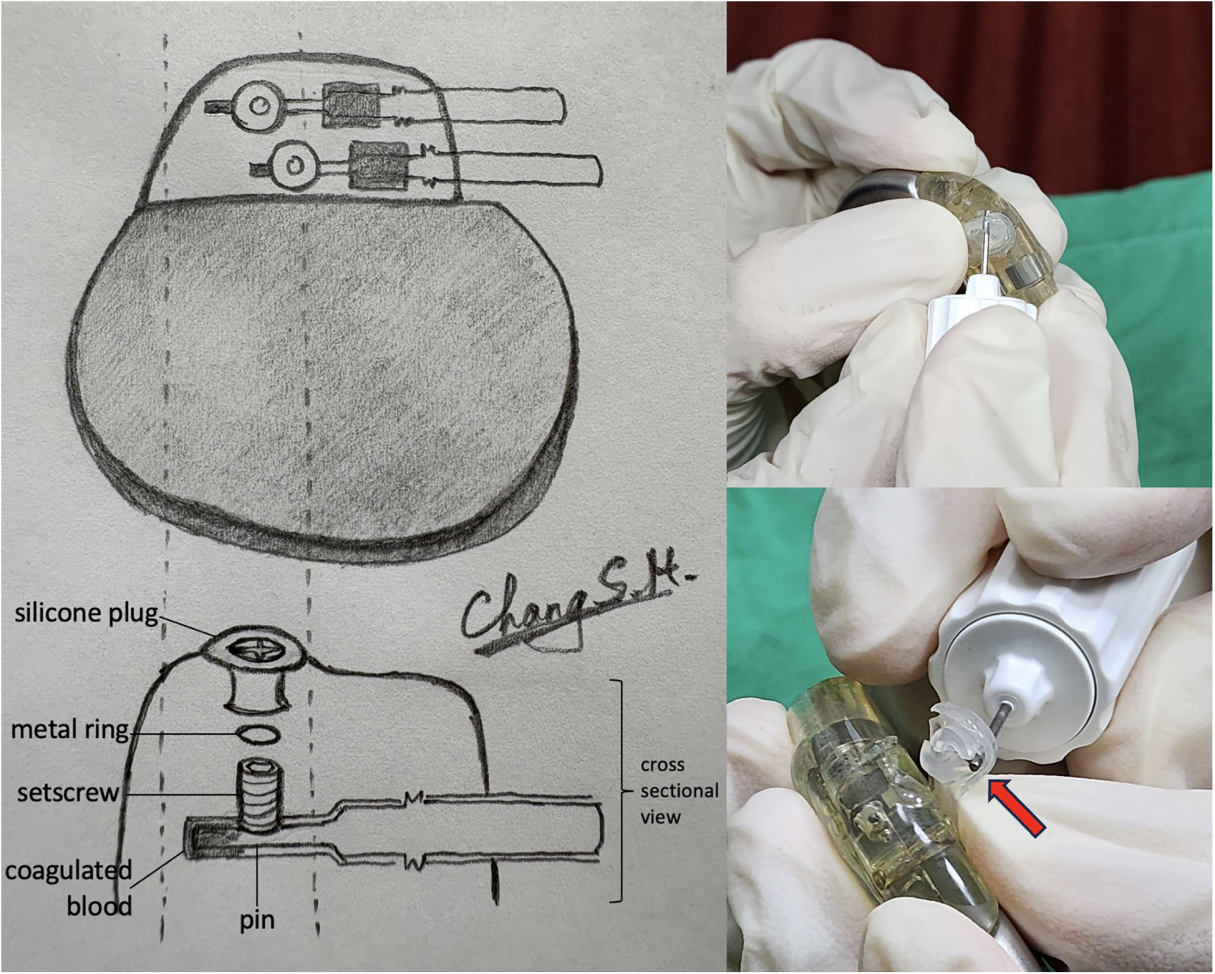
Illustration of the pacemaker connector assembly and the disruptive procedure. Upper left: normal composition of the connector, showing the silicone plug, metal ring, setscrew, and lead pin in their standard positions; Lower left: connector with coagulated blood observed in the pinhole, likely originating from the silicone plug; Right: disruption of the silicone plug using a screwdriver, demonstrating the process to expose the setscrew for lead disconnection.

**Figure 3:**
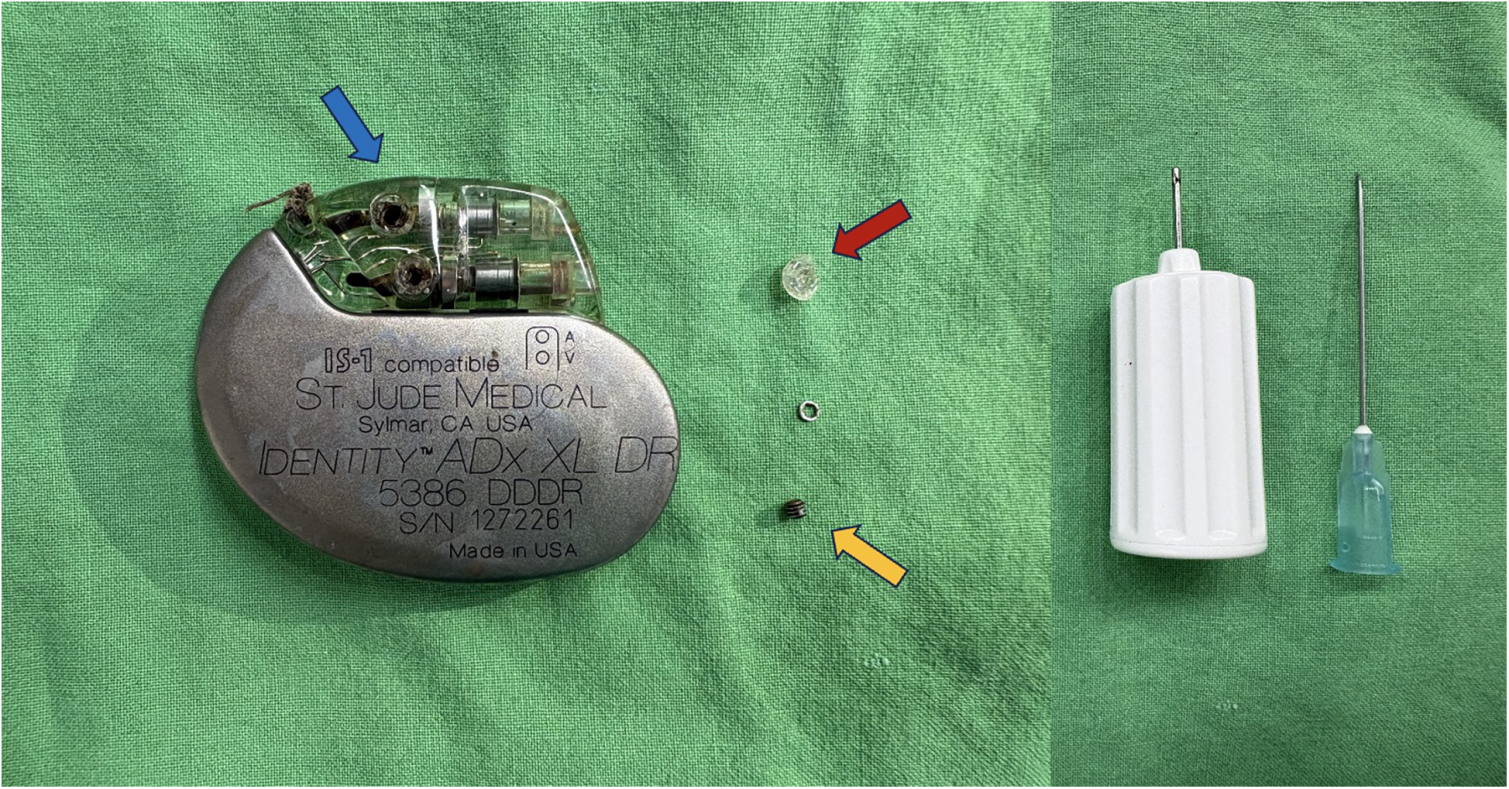
The blue arrow showed the original position of the setscrew (yellow arrow) and silicone plug (red arrow). We used a screw driver and a 22-gauge needle (right side of the figure) for removing the silicone plug and cleaned up the hexagon head of the setscrew head.

We decided to disrupt the silicone plug, and we ensured that the pacemaker incision site and pocket were carefully covered with a surgical drape prior to disrupting these parts for preventing any components loss. An illustration of the normal position of silicone plug, metal ring, setscrew, pin with coagulated blood, and the disruption procedure were provided in Figure 2. After disrupting the silicone plug, a small metal ring was exposed. Once the metal ring was removed, direct visualization of the hexagonal socket was achieved. Additional cleaning with a 22-gauge needle allowed the screwdriver to securely engage the socket, and the RA lead was successfully unscrewed (Figure 3). Upon testing, the parameters of both leads were within normal limits, with the RA lead impedance reduced to 1,175 ohms.

A new generator was connected to the leads and placed back into the pocket. The patient was discharged uneventfully the next day as planned. She returned to our outpatient clinic 13 days later, at which time all parameters remained within normal range, including an RA lead impedance of 940 ohms.

## Discussion

The incidence of lead entrapment during pulse generator replacement has been reported to range from 1.2% to 12.5% (1, 4–6). Due to lack of official experts or manufacturers guidelines and limited clinical data, operators may encounter difficulties when they are unable to disconnect the leads. The last resort to resolve this issue is to cut the lead and implant a new one. However, this approach results in an abandoned lead, prolongs the procedure time, increases the risk of infection, and compromises future magnetic resonance imaging.

Several methods have been proposed to manage “frozen lead”, including the use of orthopedic electrical drills or scalpel blades to create a new channel from the plastic head to the lead pin (1, 5, 6), bone cutters and razor saws for cutting the generator head (4, 7), and specific solvents to facilitate the disconnection of stuck leads (2). Despite these options, each method poses potential risks of lead damage and safety concerns for the operator when handling these tools. Recently, Gobeli et al. reported two successful cases using latex rubber gloves to enhance screwdriver engagement and improve torque during unscrewing, offering an easy and less destructive alternative (3).

In our case, we used a screwdriver and tweezers to remove the silicone plug and metal ring covering the setscrew, which allowed us to better understand the mechanism of the “frozen lead”. Although setscrew damage has been reported as the most common cause of “frozen lead” (3), we emphasize the importance of identifying the root cause by removing the silicone plug. In our case, some blood clots had accumulated at the head of the setscrew, deforming it and preventing proper engagement of the screwdriver. Furthermore, blood clots were also observed on the pins of both leads, which we believe contributed to the rising RA lead impedance. This was further supported by the normalization of RA impedance after we removed the clotted blood from the pin. Hybrid system with lead-generator incompatibility is another possibility to consider when sporadic impedance spikes was observed (8). To our knowledge, this is the first reported case highlighting this mechanism, and our simple technique of removing silicone plug and metal ring over the setscrew can be an effective routine first step in managing this issue.

This was our first and only case utilizing this strategy to successfully manage a “frozen lead.” The effectiveness of this approach in cases of deviated or damaged set screws requires further evaluation. Additionally, this technique may not be applicable to cases where “frozen lead” occurs due to mechanisms other than set screw damage. However, given its minimally disruptive nature, this technique could be considered if Gobeli's latex glove technique has been attempted without success, and before resorting to orthopedic electrical drills.

## Conclusion

In this case, the use of a minimally invasive technique involving the removal of the silicone plug and metal ring enabled successful detachment of a “frozen lead” without lead damage. This approach provides a valuable rescue option before progressing to more invasive options.

## Data Availability

The original contributions presented in the study are included in the article/supplementary material, further inquiries can be directed to the corresponding author.
